# SIRT1 in Neurodegenerative Diseases: Molecular Mechanisms, Disease Relevance, and Therapeutic Potential

**DOI:** 10.1096/fj.202602361RR

**Published:** 2026-09-09

**Authors:** Jiabin Duan, Jiaqi Liu, Qingsheng Meng, Jiahao Liu, Sitian Yang, Fang Zhou, Jing Cao

**Affiliations:** ^1^ Department of Human Anatomy, School of Basic Medical Sciences Zhengzhou University Zhengzhou Henan China; ^2^ The First Clinical Medical College of Zhengzhou University Zhengzhou Henan China; ^3^ Department of Anesthesiology and Perioperative Medicine People's Hospital of Zhengzhou University, Henan Provincial People's Hospital Zhengzhou Henan China; ^4^ Department of Gastroenterology The First Affiliated Hospital of Zhengzhou University Zhengzhou Henan China; ^5^ The Second Clinical Medical College of Zhengzhou University Zhengzhou Henan China; ^6^ Department of Occupational and Environmental Health, School of Public Health Zhengzhou University Zhengzhou Henan China

**Keywords:** mitochondrial dysfunction, neurodegenerative diseases, neuroinflammation, proteostasis, SIRT1, therapeutics

## Abstract

Neurodegenerative diseases are characterized by progressive protein aggregation, mitochondrial dysfunction, neuroinflammation, and cognitive decline, yet effective mechanism‐based interventions remain limited. Sirtuin 1 (SIRT1), an NAD^+^‐dependent deacetylase, has emerged as a multifunctional regulator linking stress adaptation, proteostasis, and metabolic homeostasis to disease progression. Increasing evidence indicates that SIRT1 supports cognitive resilience by coordinating synaptic plasticity, autophagy–lysosomal function, mitochondrial homeostasis, and inflammatory control. In Alzheimer's disease (AD), Parkinson's disease (PD), and Huntington's disease (HD), reduced or dysregulated SIRT1 is associated with protein aggregation, mitochondrial dysfunction, and cognitive decline, although its effects may be disease‐ and stage‐dependent, particularly in HD. This review summarizes the structural and catalytic features of SIRT1, examines the mechanisms linking SIRT1 to cognitive impairment across major neurodegenerative diseases, and evaluates the opportunities and limitations of SIRT1‐targeted therapeutic strategies.

## Graphical abstract

SIRT1, an NAD^+^‐dependent deacetylase, supports cognitive resilience by coordinating neuroprotection, synaptic plasticity, autophagy–lysosomal proteostasis, and mitochondrial energy homeostasis. Reduced or dysregulated SIRT1 is associated with protein aggregation, neuroinflammation, mitochondrial dysfunction, synaptic loss, and cognitive decline in Alzheimer's, Parkinson's, and Huntington's diseases. Pharmacological, gene‐based, and lifestyle interventions may modulate SIRT1; although therapeutic effects depend on disease context, stage, cell type, and dose.

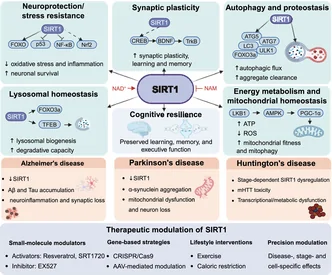

## 1 Introduction

SIRT1 is an NAD^+^‐dependent deacetylase first identified in 2000 by Imai and his research team [1]. SIRT1 is involved in various cellular processes, including energy metabolism, stress adaptation, DNA repair, and protein homeostasis regulation. It also serves as a key sensor of cellular energy status [2, 3, 4]. SIRT1 maintains metabolic and redox balance by regulating multiple signaling pathways, including AMP‐activated protein kinase (AMPK) and peroxisome proliferator‐activated receptor gamma coactivator 1 alpha (PGC‐1α), through modulation of transcription factors, histones, and non‐histone proteins [5, 6].

Recent evidence indicates that SIRT1 plays an important role in cognition‐related processes that are highly relevant to neurodegenerative disease progression. It can utilize multiple mechanisms to facilitate daily learning and memory while enhancing synaptic plasticity. Specific mechanisms include cAMP response element‐binding protein (CREB)/brain‐derived neurotrophic factor (BDNF)‐mediated transcription, regulation of mitochondrial quality, promotion of autophagy–lysosomal proteostasis, and suppression of inflammatory signaling [7, 8, 9, 10, 11, 12]. Existing studies suggest that SIRT1 expression or activity is generally reduced in AD and PD, whereas in HD its regulation appears to be more stage‐ and context‐dependent. Concurrent in vivo processes include accumulation of pathogenic proteins, neuronal dysfunction, and cognitive decline [8, 9]. This has also sparked interest in SIRT1 as a potential biomarker and therapeutic target for cognitive disorders.

Current mechanistic studies and translational research have identified numerous approaches to modulate SIRT1 activity, including pharmacological activation, gene‐based strategies, and lifestyle interventions, particularly exercise and caloric restriction (CR) [4, 10, 13, 14]. Despite broad evidence supporting a neuroprotective role for SIRT1, its effects are not uniformly beneficial across disease settings. Key unresolved issues include evidence strength across disease models, the relative importance of cognition‐proximal mechanisms, and the context dependence of SIRT1‐targeted interventions across cell types and disease stages. Here, we examine how SIRT1 links proteostasis, synaptic plasticity, metabolic resilience, and neuroinflammatory control to cognitive outcomes, and we assess the translational implications of modulating SIRT1 in AD, PD, and HD.

## 2 Structure and Function of the SIRT1 Protein

### 2.1 Core Architecture and Subcellular Distribution of SIRT1

The human SIRT1 gene is located in the q22.1 region of chromosome 10. The human SIRT1 gene contains nine exons and eight introns and encodes a protein of 747 amino acids [4]. Structurally, SIRT1 is organized around a highly conserved catalytic core domain. The N‐terminal and C‐terminal regions flanking this core exhibit relatively limited structural features [15].

The catalytic core domain of SIRT1 is composed of approximately 275 amino acids. Overall, this catalytic core exhibits an elliptical conformation and is primarily composed of two spherical subdomains connected by a cofactor‐binding loop. One is a larger Rossmann fold domain, and the other is a smaller Zn^2+^ binding domain, which together form a fissure accommodating NAD^+^ and acetylated lysine [16]. The large subdomain primarily binds to NAD^+^, while the small subdomain participates in substrate recognition through zinc finger motifs containing Cys‐X_2–4_‐Cys‐X_15–40_‐Cys‐X_2–4_‐Cys. This motif interacts with the cofactor‐binding loop, not only revealing the structural characteristics of the nicotinamide‐binding pocket but also effectively protecting the catalytic intermediate from premature hydrolysis [17]. Compared to other members of the Sirtuins family, SIRT1 exhibits a longer N‐terminal and C‐terminal unstructured tail. The N‐terminal region of SIRT1 contains nuclear localization signals (NLS) and nuclear export signals (NES), which facilitate its nuclear‐cytoplasmic shuttling [18]. Enzyme activity in this region can also be regulated through post‐translational modulation and interactions with cofactors, such as AROS [16]. In addition, the C‐terminal domain contributes to stabilizing the catalytic core conformation and enhances the efficiency of deacetylation [19].

**FIGURE 1:**
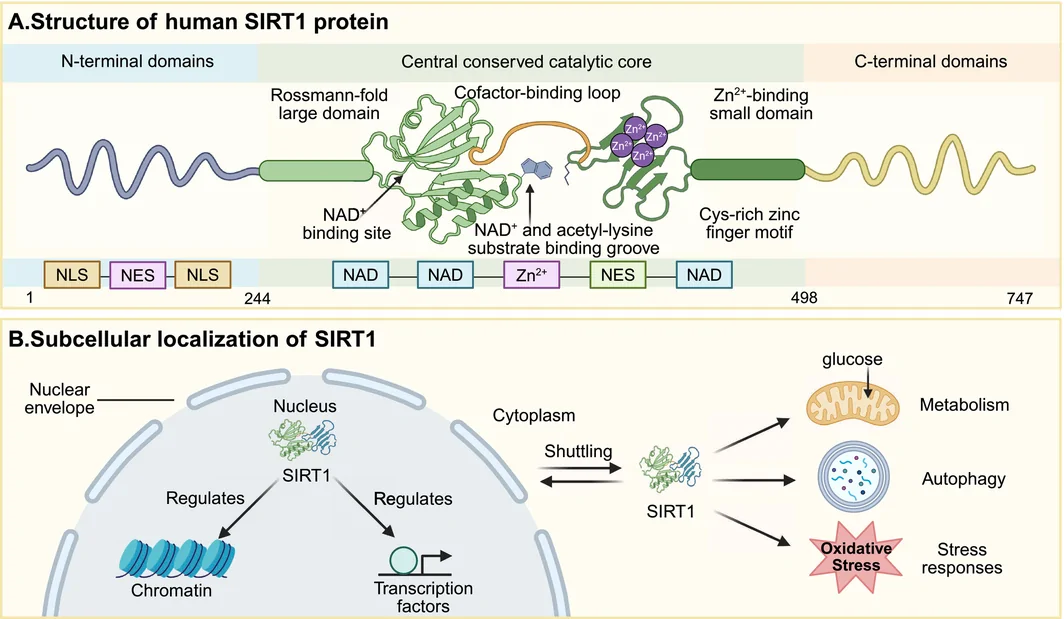
Structural organization and subcellular distribution of SIRT1. SIRT1 contains a conserved catalytic core flanked by extended N‐terminal and C‐terminal regions. The catalytic core includes a Rossmann‐fold large domain, a Zn^2+^‐binding small domain, and a cofactor‐binding loop that together form the NAD^+^ and acetyl‐lysine binding cleft. SIRT1 also contains NLS and NES motifs that support nucleocytoplasmic shuttling. In the nucleus, SIRT1 mainly regulates chromatin and transcription factors, whereas in the cytoplasm, it is more closely linked to metabolism, autophagy, and stress responses.

SIRT1 typically functions as a nuclear protein, but its subcellular localization exhibits clear cell‐type and developmental‐stage dependence [20]. For example, in renal tubular epithelial cells, podocytes, ependymal cells, and neurons in multiple brain regions, SIRT1 displays a dynamic shuttling distribution between the nucleus and cytoplasm [21, 22]. In embryonic cardiomyocytes and spermatocytes, it is primarily localized in the nucleus; whereas in adult cardiomyocytes, it can be distributed in both the nucleus and cytoplasm [22]. This spatial plasticity suggests that SIRT1 localized to the nucleus tends to regulate epigenetics by modulating the acetylation levels of histones and transcription factors, while SIRT1 localized to the cytoplasm or regions adjacent to mitochondria is more commonly involved in regulating metabolic activities, autophagy, and cellular stress responses [23, 24] (Figure 1).

### 2.2 Deacetylase Activity: The Deacetylation Mechanism of SIRT1 and Its Dependence on NAD ^+^

The structural basis of SIRT1‐mediated deacetylation is primarily located in two subdomains of its catalytic core and the cofactor‐binding loop. The larger subdomain adopts a typical Rossmann fold structure responsible for NAD^+^ binding, while the smaller subdomain contains zinc finger motifs for substrate recognition. The cofactor‐binding loop stabilizes NAD^+^ binding to prevent its dissociation, limits the formation of intermediates during the reaction process, and also reduces water ingress, thereby inhibiting side reactions. The cofactor‐binding loop can also promote product release by stabilizing conformational closure [20].

The SIRT1‐catalyzed deacetylation process can be divided into four consecutive steps. In the first step, NAD^+^ enters the active pocket of SIRT1 and binds to the acetylated lysine substrate, forming the initial complex. The nicotinamide ring interacts with the carbonyl oxygen of acetylated lysine. In the second step, the nicotinamide‐ribose glycosidic bond is cleaved, releasing free nicotinamide and generating an O‐alkyl‐ADP‐ribose intermediate. In the third step, the acetyl group from the lysine side chain is transferred to the 2′‐hydroxyl group of ADP‐ribose, forming the 2′‐O‐acetyl‐ADP‐ribose intermediate. In the final step, the reaction ultimately yields deacetylated lysine and 2′‐O‐acetyl‐ADPR [25]. The second step remains uncertain, with this uncertainty arising from the sequence of glycosidic bond cleavage and nucleophilic attack. This step can be carried out through two approaches, including single‐molecule nucleophilic substitution reactions or double‐molecule nucleophilic substitution reactions. The main difference between these two methods lies in whether a positively charged oxygen–carbon cation intermediate can be formed before the nucleophilic attack occurs. In the model of single‐molecule nucleophilic substitution reactions, the glycosidic bond is first cleaved, generating a positively charged oxygen–carbon cation intermediate, followed by subsequent nucleophilic steps. Structural studies support this cleavage‐first model. In the Hst2/carba‐NAD^+^ complex, the acetyl carbonyl oxygen forms hydrogen bonds with both the 2′‐OH and 3′‐OH groups of ribose. This interaction not only inhibits nucleophilic attack at the conformational level but also exerts inhibitory effects on molecular interactions, thereby aligning more closely with the previously proposed intermediate formation mechanism. In the model of bimolecular nucleophilic substitution reaction, the carbonyl oxygen of acetylated lysine can directly attack ribose C1′, while simultaneously breaking the glycosidic bond, with both processes occurring in concert. Structural data support this view, as the crystal structure of Sir2Tm bound to NAD^+^ and acetylated peptides demonstrates that synchronous cleavage of glycosidic bonds during nucleophilic attack can be spatially achieved [20, 26, 27].

Since the binding of NAD^+^ to acetyl‐lysine substrate serves as the starting point of the catalytic cycle, the intracellular [NAD^+^]/[NADH] ratio is continuously altered in response to changes in the redox state of cells, thereby strictly regulating the activity of SIRT1 [2, 4]. An increase in the intracellular [NAD^+^]/[NADH] ratio significantly enhances SIRT1 activity. Since NAD^+^ functions as an essential substrate, higher NAD^+^ availability can produce effects similar to allosteric activation. Similarly, nicotinamide (NAM) and NADH also act as endogenous inhibitors to regulate SIRT1 activity. Under metabolic stress conditions such as fasting or exercise, intracellular NAD^+^ levels increase, which enhances the binding capacity of SIRT1 substrates and consequently improves deacetylation efficiency. By contrast, a typical negative feedback inhibition loop involves the reaction byproduct NAM occupying the nicotinamide‐binding pocket of SIRT1 and competing with NAD^+^ for binding [28, 29]. Because intracellular NAD^+^ availability is highly sensitive to metabolic stress and mitochondrial dysfunction, this catalytic dependence places SIRT1 at a critical intersection between energy imbalance and neurodegenerative disease progression.

### 2.3 Mechanisms by Which SIRT1 Regulates Target Proteins and Its Roles in Cellular Metabolism

At the signal transduction level, SIRT1 serves as a crucial bridge between immunomodulation and metabolic remodeling. This is primarily achieved through the coordinated regulation of key signaling pathways such as inflammatory responses, cell survival status, energy metabolism activities, and autophagy by deacetylating transcription factors, histones, and various non‐histone substrates. SIRT1 exerts its effects by modulating key transcription factors, including nuclear factor κB (NF‐κB), hypoxia‐inducible factor 1‐α (HIF‐1α), tumor protein 53 (p53), and forkhead box protein O (FOXO). It not only enhances antioxidant defense and cellular stress adaptation capabilities but also effectively suppresses excessive inflammatory responses and cell death. At the epigenetic level, SIRT1 regulates chromatin structure through histone deacetylation, thereby influencing the transcriptional processes of genes associated with inflammation and metabolism. In cytoplasmic and mitochondrial environments, SIRT1 also regulates glucose and lipid metabolism, autophagy flux, and energy utilization efficiency, primarily through three pathways: modulation of the liver kinase B1‐AMP‐activated protein kinase (LKB1–AMPK) axis, autophagy‐related proteins, and proteins involved in mitochondrial function. Through these mechanisms, SIRT1 reduces the risk that excessive ROS accumulation will impair synaptic transmission and plasticity. It also helps prevent metabolite buildup from lowering energy‐production efficiency and amplifying inflammatory signaling. Together, these processes provide a molecular basis for maintaining cognitive function.

In the context of inflammation and cell survival, SIRT1 deacetylates the p65 subunit of the NF‐κB complex, directly dampening NF‐κB transcriptional activity. It also curbs NF‐κB signaling indirectly by engaging upstream or parallel pathways such as AMPK, peroxisome proliferator‐activated receptor alpha (PPARα), and PGC‐1α [30, 31]. Through these actions, SIRT1 reduces the expression of pro‐inflammatory cytokines, including interleukin‐6 (IL‐6) and tumor necrosis factor‐alpha (TNF‐α), promoting resolution of inflammation and improving cellular oxidative metabolism [32]. Prior studies link elevated NF‐κB p65 expression and activity to increased inflammatory mediators, which tend to aggravate rather than alleviate inflammatory responses [32]. Therefore, the deacetylation of NF‐κB p65 by SIRT1 should be regarded as a typical anti‐inflammatory mechanism [33]. SIRT1 can also bind to HIF‐1α and deacetylate it, suppressing HIF‐1α–dependent transcription. This shift lowers the production of pro‐inflammatory factors such as interleukin‐1 beta (IL‐1β) while relieving repression of anti‐inflammatory mediators, including interleukin‐1 receptor antagonist (IL‐1RA) and interleukin‐10 (IL‐10), thereby favoring an anti‐inflammatory microenvironment that supports tissue repair [34]. On the survival side, endogenous SIRT1 selectively deacetylates p53 at lysine 382 (K382), suppressing excessive cell cycle arrest or attenuating p53‐dependent pro‐apoptotic transcriptional programs and providing cytoprotection under inflammatory or stress conditions [35, 36, 37]. Under DNA damage or oxidative stress, nuclear SIRT1 forms complexes with the FOXO family and reshapes FOXO‐driven transcription through deacetylation, biasing the output toward antioxidant enzymes and cytoprotective genes without excessively activating pro‐apoptotic pathways, thereby limiting unnecessary cell loss [38]. In addition to NF‐κB‐centered regulation, SIRT1 may intersect with the cGAS–STING pathway, an innate immune sensor of cytosolic DNA that contributes to sterile inflammation [39]. Mitochondrial dysfunction, cytosolic DNA leakage, and oxidative stress can promote cGAS–STING activation [40]. SIRT1 could indirectly restrain cGAS–STING activation; however, direct evidence in neurodegenerative models remains limited and should be tested in a cell‐type‐ and disease‐stage‐specific manner. IL‐6 should also be interpreted in a context‐dependent manner. Transient classical IL‐6 signaling can support adaptive and regenerative responses, including exercise‐associated myokine effects, whereas persistent IL‐6 trans‐signaling is more closely associated with chronic inflammation and tissue injury [41]. Through NF‐κB deacetylation and chromatin regulation, SIRT1 may reduce sustained IL‐6 production, but whether it selectively shifts the balance between classical and trans‐signaling in the brain remains unresolved. To further place this pathway in a neuroinflammatory context, maintenance of mitochondrial integrity and mitophagic clearance by SIRT1 may reduce mtDNA release into the cytosol and thereby limit sustained cGAS–STING activation [42]. NAD^+^ availability may also influence this interface by linking SIRT1 activity with mitochondrial quality control and DNA‐damage responses. In addition, cGAS–STING signaling can shape M1‐like and M2‐like macrophage/microglial states, and persistent activation may contribute to inflammatory imbalance [39].

At the epigenetic level, SIRT1 can accumulate at specific gene promoter regions and deacetylate lysine residues such as histone H1 lysine 26 (H1K26), histone H3 lysine 9/14/18/56 (H3K9/K14/K18/K56), and histone H4 lysine 6/12/16 (H4K6/K12/K16). This activity shifts chromatin from a more open configuration toward a relatively compact state, thereby suppressing promoter activity of pro‐inflammatory genes, including TNF‐α and IL‐1β, and alleviating inflammatory injury in hepatocytes and other tissues [32]. In certain inflammatory contexts, SIRT1 further lowers transcription at cytokine promoters through histone H3 lysine 16 (H3K16) deacetylation [4]. This mode of regulation through histone deacetylation and gene silencing not only explains the anti‐inflammatory mechanism of SIRT1 at the epigenetic level but also provides insights into how SIRT1 long‐term regulates cognition‐related genes.

During the regulation of non‐histone substrates and metabolism, SIRT1 modulates the stability and activity of LKB1 through deacetylation, thereby finely regulating AMPK activation to support cellular energy‐sensing states and metabolic reprogramming. Among them, LKB1 is a classic upstream kinase in the AMPK pathway, while AMPK serves as a core regulator of energy homeostasis [43]. SIRT1 also directly regulates autophagy by deacetylating autophagy‐related proteins such as autophagy‐related 5 (ATG5), autophagy‐related 7 (ATG7), and microtubule‐associated protein 1 light chain 3 (LC3). Positioned in the nucleus, SIRT1 activates the FOXO transcription factor family, which enhances autophagy by upregulating the expression of components in the autophagy pathway, thereby initiating and maintaining an efficient autophagy flux. This pathway facilitates the clearance of damaged organelles and abnormal protein aggregates, thereby maintaining intracellular homeostasis [44].

**FIGURE 2:**
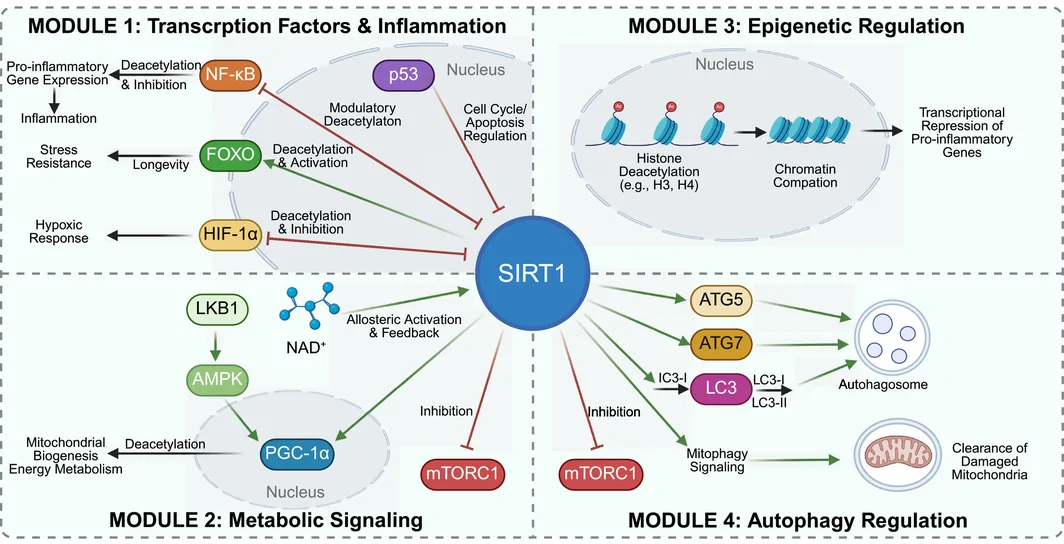
Major regulatory modules through which SIRT1 coordinates inflammation, epigenetic regulation, metabolism, and autophagy. SIRT1 modulates transcription factors involved in inflammation and cell survival, represses pro‐inflammatory gene expression through histone deacetylation, regulates metabolic signaling through the LKB1–AMPK–PGC‐1α axis, and promotes autophagy by targeting proteins such as ATG5, ATG7, and LC3. Together, these interconnected actions help maintain cellular homeostasis and support cognitive function.

Furthermore, during glucose metabolism, SIRT1 deacetylates and activates PGC‐1α, promoting the biosynthesis of mitochondria and oxidative fatty acids, thereby enhancing insulin sensitivity [45]. Under fasting conditions, hepatic SIRT1 exerts precise regulation over gluconeogenesis gene expression by deacetylating forkhead box protein O1 (FOXO1), thereby ensuring glucose production while preventing excessive gluconeogenesis [46]. Under nutritional deprivation, SIRT1 also inhibits glycolytic flux through phosphoglycerate mutase 1 (PGAM1), enabling more efficient utilization of limited energy substrates [47]. In lipid metabolism, SIRT1 deacetylates and positively regulates liver X receptor (LXR), promoting reverse cholesterol transport and high‐density lipoprotein (HDL) formation [48]. In white adipose tissue, SIRT1 deacetylates FOXO1 to increase adipose triglyceride lipase (ATGL) expression, enhancing basal lipolysis, and it also contributes to balancing lipid synthesis and breakdown by influencing factors such as sterol regulatory element‐binding protein (SREBP) [49, 50]. With respect to mitochondrial function, SIRT1 upregulates expression of electron transport chain complexes I–V through PGC‐1α, improving electron transfer efficiency, increasing ATP output, and reducing reactive oxygen species (ROS) generation [45]. At the same time, AMPK–NAD^+^–SIRT1 forms a positive‐feedback loop: AMPK activation raises NAD^+^ levels and strengthens SIRT1 activity, while SIRT1, by regulating LKB1 and downstream effectors, further promotes AMPK phosphorylation, together driving fatty acid oxidation, glucose uptake, and mitophagy [29, 51]. SIRT1 can also suppress mechanistic target of rapamycin complex 1 (mTORC1) signaling, relieving its inhibition of PTEN‐induced kinase 1/Parkin RBR E3 ubiquitin protein ligase (PINK1/Parkin)‐mediated mitophagy and promoting clearance of damaged mitochondria, thereby further improving cellular energy‐metabolic efficiency and stress resilience [52] (Figure 2). Viewed as an integrated hub, the AMPK–SIRT1–PGC‐1α axis couples cellular energy sensing to mitochondrial biogenesis, antioxidant capacity, dynamics, and mitophagy [53]. AMPK can increase NAD^+^ availability and promote catabolic adaptation, SIRT1 deacetylates PGC‐1α and other stress‐response substrates, and activated PGC‐1α coordinates nuclear programs that support respiratory‐chain function and mitochondrial renewal [54]. This reciprocal network may be especially relevant when mitochondrial quality‐control failure contributes to synaptic dysfunction. Its consequences are nevertheless disease‐ and cell‐type‐dependent, and combined strategies targeting more than one node may provide synergy but also increase the risk of excessive or mistimed pathway activation. Recent analyses further support the AMPK–SIRT1–PGC‐1α axis as a self‐reinforcing metabolic network in which AMPK‐dependent increases in NAD^+^ enhance SIRT1 activity and SIRT1‐mediated deacetylation of PGC‐1α promotes mitochondrial biogenesis and oxidative metabolism [54]. Because SIRT1 is NAD^+^ dependent, limited NAD^+^ availability during prolonged metabolic or mitochondrial stress may constrain this feedback loop. This network architecture also suggests that modulation of one node can influence the others, although sustained or excessive activation may increase context‐dependent and off‐target effects.

Collectively, SIRT1 exerts its pleiotropic functions through the deacetylation of a wide spectrum of transcription factors, epigenetic regulators, metabolic enzymes, autophagy‐related proteins, and DNA repair components, thereby integrating inflammatory control, metabolic adaptation, genome stability, and proteostasis into a unified regulatory network (Table 1).

**TABLE 1:** Representative SIRT1 deacetylation substrates and their functional consequences.

Substrate category	Substrate	Key deacetylation sites	Functional consequence	Associated biological processes	References
Transcription factor	NF‐κB p65	K310	Suppression of transcriptional activity and pro‐inflammatory gene expression	Anti‐inflammatory response, immune homeostasis	[32, 33]
Transcription factor	p53	K382	Attenuation of p53‐dependent pro‐apoptotic transcription	Cell survival, stress resistance	[35, 36, 37]
Transcription factor	FOXO1/FOXO3a	Multiple sites	Shift toward antioxidant and cytoprotective transcriptional programs	Oxidative stress resistance, cellular adaptation	[38, 55]
Epigenetic regulator	Histone H3	K9, K14, K18, K56	Chromatin condensation and transcriptional repression	Epigenetic regulation	[4, 32]
Metabolic regulator	LKB1	Multiple sites	Activation of AMPK signaling	Energy sensing and metabolic homeostasis	[43]

## 3 Mechanisms by Which SIRT1 Contributes to Cognitive Function

**FIGURE 3:**
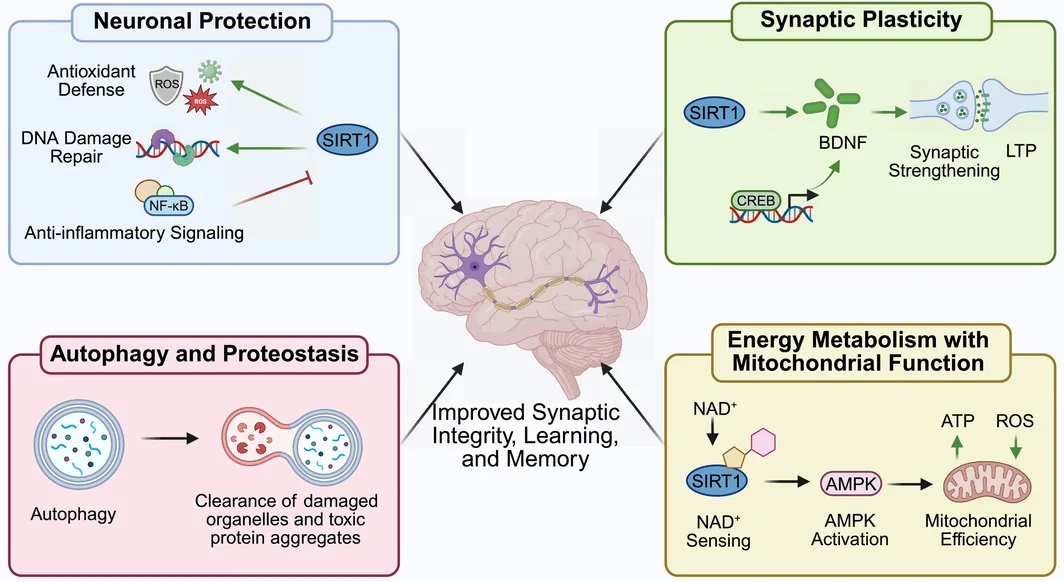
Major mechanisms through which SIRT1 influences disease‐relevant processes. SIRT1 contributes to cognitive maintenance through coordinated effects on neuroprotection, synaptic plasticity, autophagy–lysosomal proteostasis, and mitochondrial metabolic homeostasis. Through these interconnected pathways, SIRT1 helps preserve synaptic integrity and supports learning and memory.

SIRT1 does not support cognition through a single linear pathway; instead, it modulates multiple, intertwined networks. It acts mainly by protecting neurons, enhancing resistance to oxidative stress, promoting repair of DNA damage, limiting inflammation, and preserving mitochondrial function [56, 57, 58, 59, 60, 61]. SIRT1 also helps maintain and strengthen synaptic plasticity, in part by tuning pathways such as CREB/BDNF [62, 63]. In parallel, it adjusts cellular energy metabolism by sensing energy status through NAD^+^ and promoting metabolic adaptation via AMPK [4, 28, 29]. These mechanisms have been discussed above. It supports clearance of toxic species as well: by boosting autophagy, SIRT1 becomes crucial for preventing abnormal protein aggregation in neurodegenerative disease contexts [44]. Ultimately, at the epigenetic level, SIRT1 can modulate cognitive function by silencing genes that impair cognitive performance or activating genes that protect cognitive function, thereby gradually influencing cognitive outcomes over time [64]. These pathways collectively constitute the molecular basis for maintaining learning capacity, memory function, and higher cognitive functions (Figure 3).

SIRT1 exerts coordinated effects across multiple brain cell types, thereby helping maintain cognitive function. In this process, these cell types contribute distinct yet complementary protective roles (Table 2).

**TABLE 2:** Cell–type–specific roles of SIRT1 in cognitive function.

Cell type	Major SIRT1 targets	Functional outcomes	Implications for cognition	References
Neurons	FOXO, p53, CREB, PGC‐1α	Antioxidant defense, reduced apoptosis, preserved mitochondrial function	Maintenance of synaptic integrity and neuronal survival	[38, 51, 52, 53, 54]
Microglia	NF‐κB, PPARγ, TREM2, CD36	Suppression of neuroinflammation, enhanced phagocytosis	Reduced inflammation‐associated cognitive decline	[60, 61, 65, 66, 67, 68]
Astrocytes	NF‐κB, AMPK	Decreased release of pro‐inflammatory cytokines	Stabilization of neural microenvironment	[30, 60]

### 3.1 Neuroprotection

In the antioxidant defense mechanism, SIRT1 deacetylates FOXO family members such as FOXO1/3a, thereby altering their transcriptional output and promoting the transition from apoptotic programs to antioxidant cell protection programs. This change promotes the expression of antioxidant enzymes such as catalase (CAT), zinc/manganese superoxide dismutase (Zn/Mn‐SOD), peroxiredoxin 3/5 (PRDX3/5), and glutathione‐related enzymes, thereby scavenging excess ROS, reducing oxidative damage, and preventing neuronal dysfunction and synaptic plasticity impairment [55]. Nuclear factor erythroid 2‐related factor 2 (Nrf2) is a key regulator of redox homeostasis and is closely linked to transcriptional activation of many antioxidant enzymes. Existing evidence suggests that the SIRT1–Nrf2 axis participates in antioxidant defense across multiple models, forming one of the important pathways for countering oxidative stress in the brain [69]. Oxidative stress and endoplasmic reticulum (ER) stress can reinforce each other in protein‐aggregation disorders: ROS perturbs protein folding and ER redox balance, whereas a prolonged unfolded protein response (UPR) can further impair mitochondria and increase oxidative injury [70]. SIRT1 has been reported to modulate UPR‐related signaling and chaperone programs, potentially favoring adaptive proteostasis over terminal stress responses. This connection provides a mechanistic bridge among SIRT1‐dependent antioxidant defense, ER quality control, and the handling of Aβ, Tau, α‐synuclein, and mHTT. The UPR is coordinated mainly through PERK, IRE1α, and ATF6, whereas prolonged ER stress can shift this adaptive response toward apoptosis, including activation of the PERK/CHOP pathway [71]. SIRT1 may influence this balance through HSF1‐associated chaperone regulation, thereby linking ER quality control with cellular proteostasis [72]. This interaction may be particularly relevant in PD, where α‐synuclein accumulation and mitochondrial dysfunction can reinforce proteotoxic and ER stress. Similar coupling of ER stress with oxidative stress, inflammation, and protein‐degradation pathways has also been observed in other tissues [70].

In DNA damage repair, SIRT1 coordinates the capacity to repair double‐strand breaks (DSBs) and single‐strand breaks (SSBs) by deacetylating several core repair proteins, thereby supporting genomic stability in neurons. SIRT1 forms a complex with Ku70 (XRCC6, X‐ray repair cross‐complementing protein 6) and deacetylates it, strengthening Ku70 binding to DSB ends and promoting non‐homologous end joining (NHEJ), which favors rapid lesion repair [73, 74]. SIRT1 also deacetylates Nijmegen breakage syndrome protein 1 (NBS1) to stabilize the MRN complex (MRE11–RAD50–NBS1), enabling efficient recruitment of ataxia‐telangiectasia mutated (ATM) to damage sites and activation of cell‐cycle checkpoints and downstream repair cascades [75, 76]. Within base excision repair (BER), SIRT1 deacetylates X‐ray repair cross‐complementing protein 1 (XRCC1) at sites such as lysine 260/298/431 (K260, K298, and K431), modulating its interaction with beta‐transducin repeat‐containing protein (β‐TrCP) and its half‐life. This helps prevent harmful “trapping” of poly(ADP‐ribose) polymerase 1 (PARP1) on SSB intermediates and accelerates SSB repair [77, 78]. In parallel, SIRT1 deacetylates apurinic/apyrimidinic endonuclease 1 (APE1) at Lys6/7, enhancing its endonuclease activity and promoting functional coordination between APE1 and XRCC1, which further improves BER efficiency [78, 79]. Because neurons are terminally differentiated and have limited replicative capacity, this integration of DNA repair pathways by SIRT1 helps sustain long‐term neuronal survival and synaptic connectivity, providing a necessary foundation for stable learning and memory [59].

In anti‐inflammatory signaling, SIRT1 acts mainly through the SIRT1/NF‐κB axis. In astrocytes, by deacetylating the NF‐κB p65 subunit, SIRT1 suppresses its transcriptional activity, and it further attenuates NF‐κB signaling indirectly by engaging pathways such as AMPK, PPARα, and PGC‐1α. This reduces expression of pro‐inflammatory cytokines, including IL‐6 and TNF‐α, thereby limiting the sustained damage that neuroinflammation can impose on neurons and synapses [30, 60, 61]. In addition, SIRT1 deacetylates the FOXO family to increase antioxidant enzyme expression, which lowers the ROS burden and helps prevent excessive activation of the NOD‐like receptor family pyrin domain‐containing protein 3 (NLRP3) inflammasome, blunting the inflammation‐amplifying effects driven by oxidative stress [55, 80, 81]. Peroxisome proliferator‐activated receptor gamma (PPARγ), a key downstream node of the SIRT1–PGC‐1α axis, also contributes by repressing a range of pro‐inflammatory genes [82]. As a prototypical late‐stage pro‐inflammatory mediator, high‐mobility group box 1 (HMGB1) readily translocates from the nucleus to the cytoplasm and can be released extracellularly in response to inflammatory stimuli. SIRT1 interacts with HMGB1 at multiple lysine residues within its NLS region and deacetylates them, promoting HMGB1 nuclear retention and reducing its extracellular release and downstream inflammatory amplification, which further limits chronic inflammation–associated brain injury [83]. SIRT1 may additionally influence innate immune polarization in microglia and infiltrating macrophage‐like cells by coordinating NF‐κB, metabolic, and mitochondrial programs. Rather than enforcing a fixed binary phenotype, this regulation is likely to alter a continuum of inflammatory and reparative states that varies across disease stage and brain region [84].

Overall, SIRT1 enhances neuronal tolerance to metabolic and environmental stress through coordinated actions that strengthen antioxidant defenses, support DNA repair, restrain inflammation, and indirectly preserve mitochondrial function, providing a cellular “safety buffer” that helps sustain long‐term cognitive performance.

### 3.2 Maintaining and Enhancing Synaptic Plasticity

Regulation of synapse‐related proteins such as CREB and BDNF by SIRT1 represents a key mechanism that supports neuronal plasticity, learning and memory, and neuroprotection.

SIRT1‐mediated deacetylation of CREB and related mechanisms. CREB is a pivotal transcription factor that binds cAMP response element (CRE) sites in gene promoters to regulate downstream targets, including BDNF and other plasticity‐related genes, making it a core regulator of synaptic plasticity and learning and memory [85]. microRNA‐134 (miR‐134) is expressed in the brain and has been shown to negatively regulate dendritic spine formation in vitro. miR‐134 contains a binding site for Yin Yang 1 (YY1), a widely expressed and highly conserved transcription factor that can either activate or repress gene expression. SIRT1 is recruited to the YY1 DNA‐binding element and cooperates with YY1 to repress miR‐134 expression. Under basal conditions, miR‐134 restricts CREB translation; therefore, by suppressing miR‐134, SIRT1 relieves this translational constraint and increases CREB levels. CREB binds multiple BDNF promoters and plays a key role in activity‐dependent regulation of BDNF expression [63]. SIRT1 has also been shown to deacetylate CREB‐regulated transcription coactivator 1 (CRTC1) and to promote the interaction between CRTC1 and CREB [86]. CRTC1 is a CREB transcriptional coactivator that enhances CREB activity. In neural stem cells under high‐glucose conditions, SIRT1 can bind the hairy and enhancer of split‐1 (Hes‐1) promoter region and suppress CREB transcriptional activity via deacetylation; under low‐glucose conditions, CREB replaces SIRT1 at the Hes‐1 promoter and increases CREB‐driven transcription [87]. Together, these mechanisms help ensure that CREB‐dependent synaptic plasticity genes are activated or restrained on time across different energy and stress states.

**FIGURE 4:**
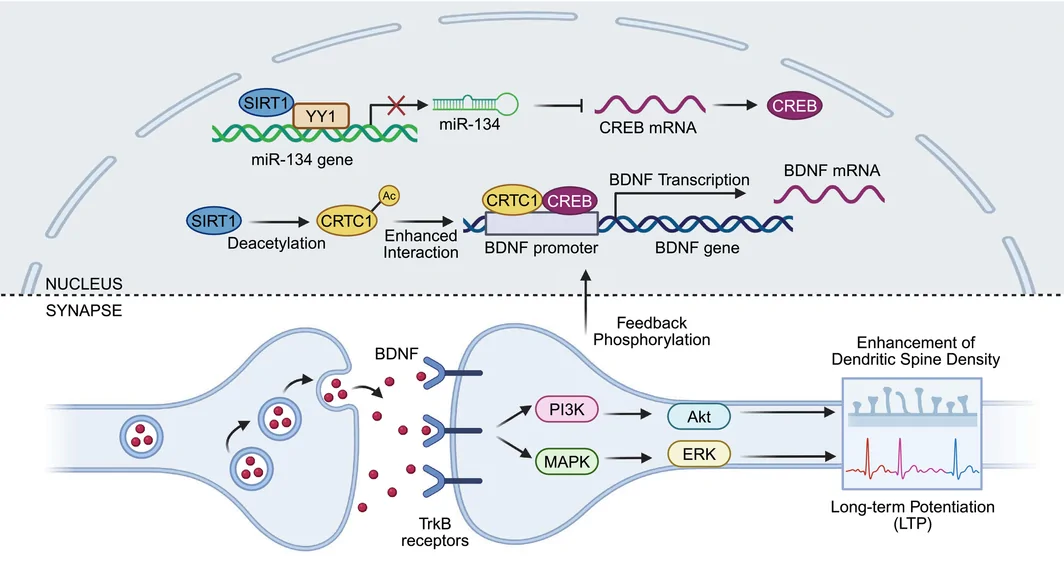
SIRT1 promotes synaptic plasticity through the CREB/BDNF signaling axis. SIRT1 cooperates with YY1 to repress miR‐134, thereby relieving translational inhibition of CREB. SIRT1 also deacetylates CRTC1 and enhances its interaction with CREB, promoting BDNF transcription. BDNF then activates TrkB downstream signaling, including the PI3K/Akt and MAPK/ERK pathways, which contribute to increased dendritic spine density and long‐term potentiation.

Regulation of the BDNF signaling pathway by SIRT1. BDNF is a key neurotrophic factor for synaptic plasticity and memory formation, playing central roles in the induction and maintenance of long‐term potentiation (LTP), pre‐ and postsynaptic structural remodeling, and the balance between excitation and inhibition [88, 89, 90, 91]. SIRT1 can enhance BDNF expression by deacetylating transcription factors. SIRT1 directly deacetylates FOXO family members, promoting FOXO binding to the BDNF promoter and thereby upregulating BDNF transcription [92]. SIRT1 also deacetylates the CREB coactivators CREB‐binding protein (CBP) and/or E1A‐binding protein 300 (p300), altering their interactions with CREB and other transcription factors and facilitating CREB‐driven activation of BDNF transcription [93]. In addition, SIRT1 deacetylates histones near the BDNF promoter region, such as histone H3 lysine 9 (H3K9) and histone H4 lysine 16 (H4K16), to shift chromatin state and, indirectly, or under specific stimuli, promote activation of particular BDNF promoters (e.g., promoter IV). SIRT1 further deacetylates the repressor methyl‐CpG‐binding protein 2 (MeCP2), relieving repression at BDNF promoter IV [11]. SIRT1 also modulates signaling downstream of the BDNF receptor. BDNF primarily signals through its high‐affinity receptor TrkB. After BDNF binds to its receptor TrkB, on one hand, it activates the phosphatidylinositol 3 (PI3K)/Akt (PKB, kinase/protein kinase B) pathway, promoting protein synthesis and local translation, which is beneficial for the development, growth, and increased density of dendritic spines, and supports the late phase of LTP [94, 95]. On the other hand, it activates the mitogen‐activated protein kinase (MAPK)/extracellular signal‐regulated kinase (ERK) pathway, which contributes to the formation and maintenance of LTP [96]. By inhibiting PTEN (phosphatase and tensin homolog) and modulating the Ras–ERK cascade, SIRT1 enhances extracellular signal‐regulated kinase 1 and 2 (ERK1/2) activation, which promotes CREB phosphorylation and expression of synaptic plasticity genes [97, 98] (Figure 4).

Through these combined actions, deacetylation‐based regulation of CREB and multilayer control of the BDNF–TrkB signaling axis, SIRT1 can coordinate LTP strength, dendritic spine density, and synaptic transmission efficiency across brain regions and developmental stages, providing structural and molecular support for learning and memory. Beyond changes in gene expression, these pathways provide a direct link between SIRT1 and neuronal plasticity at several levels: they support activity‐dependent LTP, dendritic spine formation and stabilization, local protein synthesis, presynaptic vesicle handling, and the maintenance of excitation–inhibition balance. SIRT1‐dependent metabolic and mitochondrial support is also required to meet the energetic demands of synaptic remodeling, while its anti‐inflammatory and proteostatic actions protect plasticity‐related signaling from chronic stress. These convergent effects explain why altered SIRT1 activity can influence both memory encoding and the long‐term stability of neural circuits.

### 3.3 Clearing Toxic Species Through Autophagy and Proteostasis

The clearance of abnormal protein aggregates and damaged organelles synergistically maintains cognitive function through neuronal protection and synaptic plasticity. SIRT1 maintains neuronal protein homeostasis by regulating the autophagy‐lysosomal system and chaperone networks at multiple levels, thereby reducing the accumulation of early toxic substances in neurodegenerative diseases such as AD and PD [99, 100, 101, 102, 103].

At the level of macroautophagy, SIRT1 directly deacetylates the core autophagy proteins ATG5, ATG7, and LC3, shifting them from an acetylation‐associated inhibitory state to a deacetylation‐associated active state, which favors autophagosome formation and maturation [104, 105, 106]. The acetylation–deacetylation cycle of LC3 influences its nuclear–cytoplasmic distribution; deacetylation at K49 and K51 promotes LC3 relocalization to the cytoplasm and anchoring to autophagic membranes, supporting effective autophagic flux [104, 106]. Nuclear SIRT1 can also activate transcription factors such as FOXO3a to increase expression of autophagy‐related genes, including LC3 and BCL2/adenovirus E1B 19 kDa protein‐interacting protein 3 (Bnip3), thereby enhancing autophagic flux at the transcriptional level [105, 107, 108]. SIRT1 further deacetylates the autophagy‐initiating kinase unc‐51‐like autophagy activating kinase 1 (ULK1), promoting functional assembly of the complex with RB1‐inducible coiled‐coil 1 (RB1CC1)/focal adhesion kinase family interacting protein of 200 kDa (FIP200), ATG13, and ATG101, and thereby fine‐tuning autophagy initiation [104].

In chaperone‐mediated autophagy and lysosomal function, SIRT1 deacetylates heat shock cognate 70 kDa protein (HSC70), enhancing its binding to pathogenic proteins and promoting chaperone‐mediated autophagy (CMA) and chaperone‐assisted selective autophagy (CASA), which facilitates degradation of large aggregates [109]. Meanwhile, SIRT1 deacetylates the transcription factor EB (TFEB), promoting its nuclear entry and binding to coordinated lysosomal expression and regulation (CLEAR) elements. This upregulates lysosome‐related genes such as lysosome‐associated membrane protein 2A (LAMP2A), lysosome‐associated membrane protein 1 (LAMP1), cathepsin D (CTSD), and chloride voltage‐gated channel 7 (CLCN7), thereby enhancing lysosomal biogenesis and acidification. In parallel, HSC70 recognizes KFERQ‐containing substrates and delivers them to the CMA machinery, further strengthening the degradation of pathogenic proteins such as amyloid‐β (Aβ) [110, 111, 112]. The SIRT1‐regulated autophagy–lysosome system also intersects with broader stress and regulated‐cell‐death networks. Defective autophagic flux can promote lipid peroxidation, inflammasome activation, and the accumulation of damaged mitochondria, thereby sensitizing cells to ferroptotic and pyroptotic injury [113]. Autophagy can also interact with pyroptosis and other regulated‐cell‐death pathways, and excessive or incomplete autophagy may become maladaptive [114]. SIRT1 may therefore function as a context‐dependent coordination node linking proteostasis to redox control and inflammatory cell death rather than as a uniformly pro‐autophagic switch.

**FIGURE 5:**
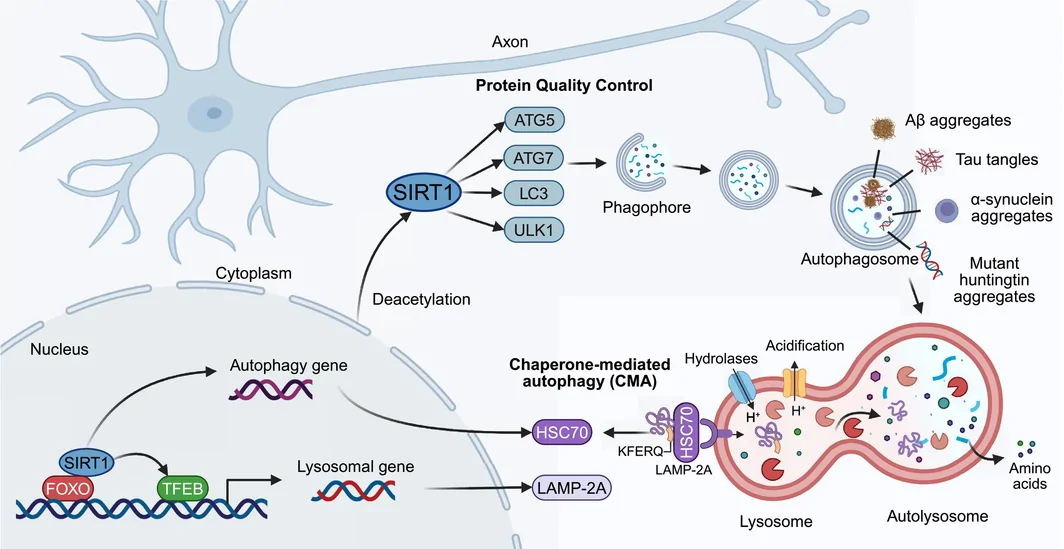
SIRT1 promotes neuronal proteostasis through macroautophagy and chaperone‐mediated autophagy. SIRT1 enhances macroautophagy by deacetylating core autophagy proteins, including ATG5, ATG7, LC3, and ULK1, thereby promoting autophagosome formation and maturation. SIRT1 also supports chaperone‐mediated autophagy and lysosomal function through HSC70 and TFEB‐related pathways, increasing lysosomal capacity and degradation of toxic protein aggregates such as Aβ, Tau, α‐synuclein, and mutant huntingtin.

These regulatory effects on the autophagy–lysosome system and molecular chaperone networks, together with the antioxidant, DNA repair, anti‐inflammatory, and metabolic reprogramming actions described above, constitute key pillars through which SIRT1 maintains neuronal homeostasis and cognitive function. In early disease stages, stronger cellular clearance capacity can delay the emergence of toxic aggregates such as Aβ, microtubule‐associated protein tau (Tau), and α‐synuclein. On the other hand, during disease progression, by preserving metabolic and protein homeostasis, SIRT1 can slow functional deterioration of synapses and neural circuits, providing a mechanistic basis for the association between SIRT1 and cognitive impairment, particularly in AD, PD, and HD, discussed in Section 4 (Figure 5).

## 4 Links Between SIRT1 and Cognitive Impairment

Neurodegenerative disorders are among the major causes of cognitive impairment in middle‐aged and older adults, and AD, PD, and HD are three representative examples. These disorders differ in etiology, early manifestations, and neuropathological features, yet all are commonly associated with progressive and multidimensional cognitive decline involving learning and memory, executive function, attention, and visuospatial ability. They also share several overlapping molecular abnormalities, including pathological protein accumulation, autophagy–lysosome dysfunction, mitochondrial damage, chronic neuroinflammation, and epigenetic dysregulation. Increasing evidence suggests that these pathological changes are often accompanied by reduced SIRT1 expression or enzymatic activity, and that the extent of this reduction is closely associated with pathogenic protein burden, brain atrophy, and the rate of cognitive decline. In contrast, moderate elevation of SIRT1 activity may delay neural circuit degeneration and cognitive deterioration through several mechanisms, including regulation of Aβ/Tau or α‐synuclein metabolism, improvement of mitochondrial function, enhancement of autophagy, and suppression of inflammatory signaling [115, 116]. In summary, investigating the expression changes of SIRT1 in AD, PD, and HD and their impact on major pathological pathways can further elucidate the common molecular basis of cognitive impairment from a cross‐disciplinary perspective, thereby facilitating the development of preventive and intervention strategies targeting SIRT1. This generalization has exceptions. In HD, the effect of SIRT1 is strongly dependent on disease stage, cell type, brain region, substrate availability, and the degree of activation. Accordingly, either carefully titrated enhancement or selective inhibition has shown benefit in different experimental settings, and SIRT1 depletion or inhibition should not be considered uniformly detrimental across all neurodegenerative contexts.

Although AD, PD, and HD exhibit significant differences in etiology and clinical manifestations, their typical features include protein homeostasis disruption, mitochondrial dysfunction, and chronic neuroinflammation, all of which are accompanied by reduced SIRT1 activity. This reflects the overlapping pathological characteristics (Table 3).

**TABLE 3:** Comparative roles of SIRT1 in major neurodegenerative diseases.

Disease	SIRT1 expression/activity	Key downstream targets	Major downstream effects of SIRT1 modulation	Cognitive endpoint	References
AD	Decreased	ADAM10, BACE1, Tau, TFEB	Amyloidogenic processing, Aβ clearance, Tau acetylation/aggregation	Memory impairment, Executive function	[14, 116, 117, 118, 119, 120, 121, 122, 123, 124]
PD	Decreased	HSF1, HSP70	α‐synuclein proteostasis, mitochondrial dysfunction, oxidative stress	Executive function, Visuospatial abilities	[125, 126, 127, 128, 129, 130, 131]
HD	Stage‐dependent decrease	CREB, PGC‐1α, FOXO	mHTT‐associated transcriptional dysregulation, mitochondrial metabolism, proteostasis stress	Executive function, Working memory	[115, 132, 133, 134, 135, 136, 137, 138, 139, 140, 141]

### 4.1 Alzheimer's Disease

AD is a neurodegenerative disorder marked by progressive cognitive and functional decline. Its core pathology includes deposition of Aβ, abnormal aggregation of Tau, and the accompanying neuroinflammation and synaptic loss. Multi‐layer brain proteomics and autopsy studies report that SIRT1 levels drop markedly in the cortex and hippocampus of patients with AD, and this decrease shows a negative correlation with neurofibrillary tangle (NFT) burden, disease duration, and the severity of cognitive impairment [116]. SIRT1 levels are also reduced in peripheral blood and serum in individuals with AD and correlate closely with neuropsychological scale scores, suggesting that SIRT1 downregulation may span the full course of AD, from the preclinical stage to dementia [117, 118].

#### 4.1.1 SIRT1 and Core Aβ/Tau‐Related Pathways

Aβ metabolism. The Aβ cascade hypothesis proposes that amyloid deposition initiates AD pathogenesis [119]. SIRT1 can lower Aβ levels by suppressing amyloidogenic production. Amyloid precursor protein (APP) undergoes two mutually exclusive processing routes. In the amyloidogenic pathway, APP is cleaved by β‐secretase and γ‐secretase to generate soluble amyloid precursor protein‐β (sAPPβ), Aβ, and amyloid precursor protein intracellular domain (AICD). In the non‐amyloidogenic pathway, APP is sequentially processed by α‐secretase and γ‐secretase, producing sAPPα (a soluble, neuroprotective fragment), p3 (a 3 kDa peptide fragment generated by α‐ and γ‐secretase cleavage of APP), and AICD [120]. On one hand, SIRT1 deacetylates retinoic acid receptor beta (RARβ) and enhances its transcriptional activity, thereby increasing expression of the α‐secretase ADAM10 (a disintegrin and metalloproteinase domain‐containing protein 10) [121]. Under basal conditions, RARβ forms a heterodimer with retinoid X receptor alpha (RXRα). Acetylation of RARβ at specific lysine residues renders this complex relatively inactive and weakens its binding to the ADAM10 promoter. SIRT1 can be recruited to the ADAM10 promoter and directly deacetylates RARβ, activating the RARβ/RXRα transcriptional complex and strengthening its drive on ADAM10 transcription. This increases α‐secretase activity, shifts APP processing toward the non‐amyloidogenic pathway, promotes the generation of sAPPα, and limits the production of full‐length Aβ peptides, thereby reducing Aβ formation [122, 123]. On the other hand, SIRT1 directly deacetylates PPARγ at K268 and K293 and deacetylates PGC‐1α, enhancing their transcriptional activity [124, 142]. Activated PPARγ and PGC‐1α form a complex that binds the peroxisome proliferator‐activated receptor response element (PPRE) within the beta‐site APP‐cleaving enzyme 1 (BACE1) promoter and recruits the nuclear receptor corepressor (NCoR), thereby repressing BACE1 transcription [122, 124]. Because BACE1 is the rate‐limiting enzyme for Aβ generation, reduced BACE1 expression shifts APP processing away from the amyloidogenic route and lowers Aβ production.

SIRT1 can also reduce Aβ burden by promoting Aβ clearance, mainly through two routes: activating the autophagy–lysosome pathway to degrade Aβ directly and enhancing microglial phagocytosis to remove extracellular Aβ. For the autophagy–lysosome route, SIRT1 first deacetylates the transcription factor TFEB, mainly at K116, enabling rapid nuclear entry and binding to CLEAR elements in promoters of lysosomal/autophagy genes. This turns on genes such as LAMP1 and CTSD [143, 144], whose products work together to support Aβ uptake, acidification, and degradation. As lysosome abundance and acidification capacity rise, microglial uptake–degradation of fibrillar Aβ accelerates, leading to reduced brain Aβ levels [145, 146]. In parallel, SIRT1 deacetylates core autophagy proteins ATG5, ATG7, and LC3; because acetylation suppresses their function, SIRT1‐driven deacetylation activates them, promotes autophagosome formation, and facilitates sequestration of Aβ into autophagosomes for lysosomal degradation after fusion [147]. SIRT1 can further deacetylate the coactivator PGC‐1α or FOXO3a, enhancing FOXO nuclear entry and upregulating autophagy‐related genes such as LC3 and Bnip3, thereby increasing autophagic flux at the transcriptional level [148]. Enhancing microglial phagocytosis provides another clearance mechanism. Microglia are resident immune cells in the brain that recognize, engulf, and degrade extracellular Aβ aggregates via phagocytosis. This process relies on surface receptors such as triggering receptor expressed on myeloid cells 2 (TREM2) and cluster of differentiation 36 (CD36) to bind Aβ. SIRT1 deacetylates and activates PPARγ, and because the CD36 promoter contains a PPAR response element, activated PPARγ can bind directly to the CD36 promoter and drive its transcription and protein expression [65, 66]. PPARγ activation also markedly increases TREM2 expression [67]. Higher levels of CD36 and TREM2 on the membrane improve microglial recognition and binding of Aβ aggregates, facilitating phagocytosis [60]. In addition, by suppressing the NF‐κB pathway as described above, SIRT1 reduces the release of pro‐inflammatory cytokines [68]. Since chronic neuroinflammation impairs microglial phagocytic capacity, a more anti‐inflammatory milieu helps keep microglia in a healthier surveillance state and supports their ability to clear Aβ.

In healthy neurons, Tau is a major microtubule‐associated protein that maintains microtubule stability, promotes tubulin assembly, and supports intracellular transport. During the progression of AD, the Tau protein exhibits abnormal hyperphosphorylation, accompanied by a significant decline in its binding capacity to microtubules. This leads to impaired microtubule stability, resulting in corresponding deterioration of axonal transport function, which subsequently triggers early synaptic dysfunction and cognitive decline [149]. After dissociating from microtubules, hyperphosphorylated Tau forms soluble oligomers that directly impair synaptic plasticity and disturb neurotransmission, especially at glutamatergic synapses involved in learning and memory. These soluble oligomers further aggregate into insoluble fibrils and eventually develop into neurofibrillary tangles (NFTs) within neurons [150]. Pathological Tau also damages mitochondrial function, leading to neuronal energy failure and excessive production of reactive oxygen species, which further accelerates cell death. Moreover, pathological Tau, particularly oligomeric species, spreads along established neural connectivity pathways, inducing misfolding and aggregation of endogenous Tau in recipient neurons [151]. Tau itself undergoes acetylation, and Tau acetylation weakens its binding to microtubules while promoting oligomerization and NFT formation [152]. Under physiological conditions, SIRT1 can directly interact with Tau and deacetylate specific lysine residues such as K174, K274, and K280, thereby lowering acetylated Tau levels [14]. Because acetylated Tau (ac‐Tau) binds microtubules more poorly, it more readily aggregates into tangles and can accelerate trans‐synaptic spread of Tau pathology. SIRT1‐mediated deacetylation can, on the one hand, restore Tau–microtubule association and help preserve cytoskeletal stability. On the other hand, acetylation at a given lysine prevents ubiquitination at the same site, allowing Tau to evade proteasomal degradation [153, 154]. By removing acetyl groups, SIRT1 restores ubiquitin acceptor capacity at these residues, promotes K48‐linked ubiquitination of Tau, and facilitates delivery to the 26S proteasome for degradation [155]. This, in turn, reduces pathological Tau accumulation and aggregation [156, 157].

#### 4.1.2 Diagnostic Potential and Therapeutic Targeting

Diagnostic relevance. In brain tissue, SIRT1 levels decline progressively with increasing Braak stage and show a negative association with NFT burden and disease duration, suggesting value as a histological indicator of late‐stage pathological progression in AD [158, 159]. Peripheral serum SIRT1 levels are significantly lower in patients with AD than in age‐matched controls, suggesting its potential as a peripheral biomarker for early identification of AD. When considered alongside cerebrospinal fluid “gold‐standard” measures such as Aβ42, the Aβ42/40 ratio, and total tau protein/phosphorylated tau protein (t‐tau/p‐tau) [117], SIRT1 may serve as a complementary, possibly longitudinal, marker that reflects upstream regulatory states of the Aβ/Tau axis, aiding patient stratification and monitoring of treatment response.

Therapeutic implications. Synthetic small‐molecule activators such as SRT1720 and SRT2104 exhibit higher potency and superior pharmacokinetic stability. These activators not only activate the SIRT1‐AMPK‐Nrf2 antioxidant pathway but also inhibit NF‐κB‐mediated neuroinflammatory responses. They have been demonstrated to improve learning and memory abilities while reducing neuronal apoptosis [160, 161]. In addition to pharmacological interventions, CR, intermittent fasting (IF), and aerobic exercise can increase SIRT1 levels in the central nervous system and peripheral tissues, enhance hippocampal BDNF expression, and improve synaptic plasticity. These results provide experimental evidence for cognitive protection strategies involving SIRT1 [158, 162].

### 4.2 Parkinson's Disease

PD is a progressive neurodegenerative disorder characterized by degeneration of dopaminergic neurons in the substantia nigra, striatal dysfunction, and the formation of Lewy bodies. Clinically, PD presents with prominent motor symptoms, including resting tremor, bradykinesia, rigidity, and postural or gait instability. Cognitive dysfunction is also a major non‐motor manifestation of PD. Some patients exhibit early deficits in attention, executive function, information‐processing speed, and visuospatial ability. As the disease progresses, approximately 30%–40% of patients develop Parkinson's disease dementia (PDD) [163]. This subcortical cognitive dysfunction is closely associated with impaired cortico‐striatal circuitry and is characterized by deficits in planning, cognitive flexibility, working memory, and the handling of complex tasks. In PD models, as well as in brain tissue and peripheral blood from patients, SIRT1 levels are often reduced and are closely associated with α‐synuclein aggregation, mitochondrial dysfunction, and enhanced inflammatory responses.

#### 4.2.1 SIRT1, α‐Synuclein Aggregation, and the Autophagy–Mitochondria Axis

α‐Synuclein is abundantly expressed at presynaptic terminals in the central nervous system. Under physiological conditions, it contributes to synaptic plasticity, neurotransmitter release, and vesicle trafficking. In PD, however, α‐synuclein misfolds, oligomerizes, and ultimately assembles into insoluble fibrils, driving core pathological progression [125, 126]. Pathological α‐synuclein can be released from affected neurons, taken up by neighboring healthy neurons, and then seed pathological conversion of endogenous α‐synuclein in the recipient cell [127]. SIRT1 does not appear to modify α‐synuclein directly. Instead, it suppresses pathological α‐synuclein aggregation and toxicity through two complementary mechanisms: the deacetylation–HSF1–HSP70 axis and enhancement of the autophagy–lysosome pathway. SIRT1 deacetylates heat shock factor 1 (HSF1) and strengthens its DNA‐binding capacity. By maintaining HSF1 in a deacetylated, DNA‐binding–competent state, SIRT1 prolongs HSF1 occupancy at heat shock promoters, increasing heat shock protein 70 (HSP70) expression. As a molecular chaperone, HSP70 can recognize α‐synuclein oligomers and facilitate their clearance, so this HSF1–HSP70 route provides an indirect means for SIRT1 to promote α‐synuclein removal [128, 129]. Autophagy–lysosome function is another major clearance route that delivers diverse cargo, including misfolded proteins, to lysosomes for degradation via microautophagy, macroautophagy, and chaperone‐mediated autophagy. SIRT1 enhances autophagy–lysosome activity by engaging autophagy‐related signaling pathways such as AMPK, FOXO1, and mTOR, thereby increasing cellular autophagic capacity [130]. With stronger autophagy, α‐synuclein aggregates can be removed more efficiently. In addition, in PD brain tissue, SIRT1 counteracts oxidative stress through multiple mechanisms to prevent the development of endothelial dysfunction. SIRT1 can also exert neuroprotective effects in PD by suppressing inflammation and improving mitochondrial function. Apoptosis is considered a final common pathway for the loss of dopaminergic neurons (DN) in the substantia nigra (SN) in PD and other degenerative brain diseases, and SIRT1 can inhibit the development and progression of PD neuropathology by attenuating apoptotic processes [131].

#### 4.2.2 Diagnostic Potential and Therapeutic Targeting

Diagnostic relevance. In PD, both SIRT1 mRNA and protein levels are significantly reduced in peripheral blood mononuclear cells, and this reduction correlates with disease stage and the severity of cognitive impairment, supporting SIRT1 as a potential peripheral biomarker for PD and PDD [131]. Within “oxidation–inflammation” scoring systems that integrate oxidative‐stress and inflammatory indices, SIRT1 can also serve as a key factor reflecting the systemic balance between inflammation and anti‐inflammatory capacity, offering a low‐cost and repeatable indicator for early PD detection and longitudinal disease monitoring [164, 165, 166, 167].

Therapeutic implications. Animal studies suggest that resveratrol and several synthetic SIRT1 activators can increase brain SIRT1 activity, reduce α‐synuclein aggregation and loss of nigral neurons, improve motor symptoms, and, to some extent, enhance performance in learning, memory, and executive function tasks [168, 169, 170]. Regular aerobic exercise and moderate CR also provide useful supplements for pharmacological methods. These behaviors can increase the NAD^+^/NADH ratio, thereby activating the SIRT1‐AMPK‐PGC‐1α axis and improving mitochondrial function and enhancing synaptic plasticity [171, 172]. In the future, the combination of SIRT1 activation with α‐synuclein immunotherapy, autophagy enhancers, or metabolic regulators can synergistically slow down the decline of PD‐related cognitive function.

### 4.3 Huntington's Disease

HD is an autosomal dominant neurodegenerative disorder caused by the expansion of the CAG trinucleotide repeats in the HTT gene. It typically presents with progressive choreiform involuntary movements, psychiatric symptoms, and gradually worsening cognitive impairment. Unlike AD, which is dominated by cortical memory deficits, early cognitive impairment in HD is more “subcortical,” commonly featuring psychomotor slowing and deficits in executive function, attentional control, and working memory before episodic memory, language, and social cognition become progressively involved. Imaging and pathological evidence indicate marked atrophy and neuronal loss in vulnerable regions of HD, such as the striatum and frontal cortex. At the molecular level, alterations in SIRT1 expression/activity in these regions correlate with disease course and functional decline, suggesting that SIRT1 may participate in key disease‐related pathological processes. Ultimately, the central pathogenic driver of HD is considered to be closely linked to the neurotoxic effects of mutant huntingtin (mHTT).

#### 4.3.1 Multitarget Deacetylation by SIRT1 Counteracts mHTT Toxicity

In animal models of HD and in serum or brain tissue from patients, SIRT1 expression is typically reduced [115].

The fundamental driver of HD is the expression and aggregation of mHTT. mHTT readily forms nuclear and cytoplasmic inclusions and aberrantly binds a range of transcription factors and coactivators, leading to broad transcriptional repression, including downregulation of neurotrophic and mitochondrial regulatory genes such as BDNF and PGC‐1α [132, 133, 134]. Against this background, SIRT1 provides a degree of functional “counterbalance.” On one hand, SIRT1 deacetylates CREB‐related transcriptional cofactors and coactivators such as TORC1/CRTC1, strengthening CREB‐driven transcription at the BDNF promoter. This increases BDNF expression and axonal transport along the cortico–striatal pathway, supporting striatal neuron survival and synaptic plasticity [115]. On the other hand, SIRT1 deacetylates and activates PGC‐1α, helping restore expression of genes involved in mitochondrial biogenesis and oxidative phosphorylation, and improving the mitochondrial energy deficits and excessive ROS production commonly observed in HD. In addition, by deacetylating cell‐fate regulators such as p53 and FOXO, SIRT1 reduces pro‐apoptotic gene expression, enhances stress tolerance in compromised neurons, and slows progressive apoptosis in striatal and cortical neurons [135]. At the level of proteostasis, mHTT aggregates are not only difficult to degrade but also sequester multiple protein quality‐control factors into inclusions, further weakening chaperone systems and the autophagy–proteasome machinery [136]. SIRT1 deacetylates HSF1 to promote expression of molecular chaperones such as HSP70, enhancing recognition, refolding, and targeted degradation of misfolded proteins [137]. At the same time, by modulating the acetylation status of FOXO, TFEB, and core autophagy proteins, SIRT1 may strengthen autophagic flux and lysosomal function, facilitating clearance of mHTT aggregates and associated damaged organelles [138, 146]. Although evidence for SIRT1 regulation of autophagy and inflammation in HD remains less detailed than in AD and PD, experimental work suggests that moderate enhancement of SIRT1 activity can improve energy metabolism and antioxidant defense while supporting toxic protein clearance, with potential value for slowing deterioration of HD‐related behavioral and cognitive phenotypes.

#### 4.3.2 Diagnostic Potential and Therapeutic Targeting

**FIGURE 6:**
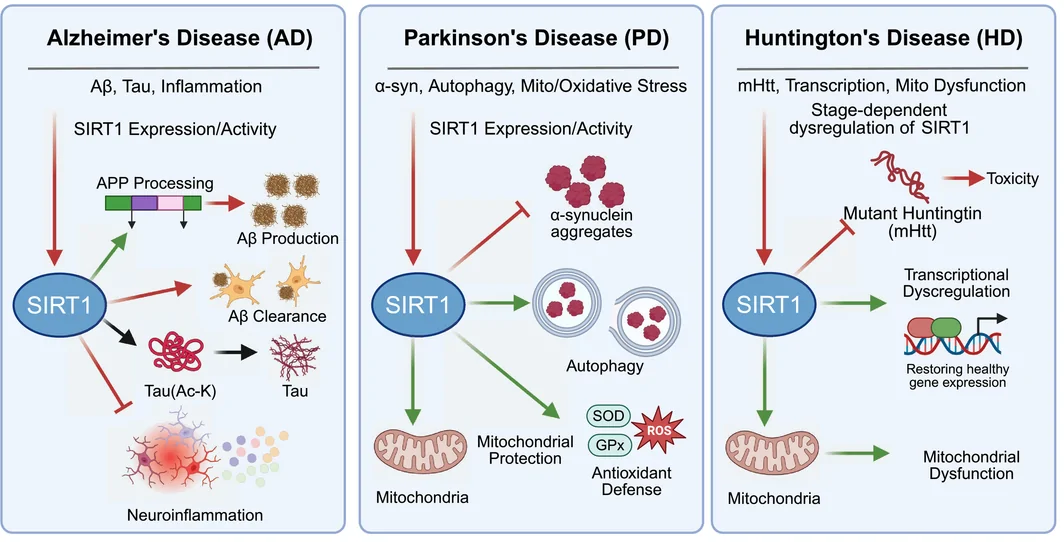
Comparative roles of SIRT1 in major neurodegenerative diseases associated with cognitive impairment. In Alzheimer's disease, reduced SIRT1 is linked to Aβ and Tau pathology, impaired Aβ clearance, and neuroinflammation. In Parkinson's disease, reduced SIRT1 is associated with α‐synuclein aggregation, autophagy impairment, mitochondrial dysfunction, and oxidative stress. In Huntington's disease, altered SIRT1 activity is linked to mHTT toxicity, transcriptional dysregulation, and mitochondrial dysfunction. Together, these disease‐specific effects highlight a shared role of SIRT1 in proteostasis, inflammation, and neuronal survival.

Interventions targeting SIRT1 in HD seem to be highly context‐dependent. Several animal studies have shown that SIRT1 overexpression or long‐term non‐selective activation may aggravate some abnormal transcription in specific disease stages, while moderate enhancement of SIRT1 activity in specific brain regions or cell types consistently shows neuroprotective effects. In some models, highly selective SIRT1 inhibitors can alleviate mHTT‐related transcriptional disorders, suggesting that the role of SIRT1 in HD may be dual and present different states with the change of disease stage and cell microenvironment [139, 140, 141]. These observations suggest that SIRT1‐based therapy in HD cannot be reduced to simple systemic activation or inhibition, but instead requires context‐aware and precisely titrated modulation. Changes in central and peripheral SIRT1 levels, together with downstream target‐gene expression, may serve as candidate markers of disease progression and pharmacodynamic response. Based on the stratification strategy based on genotype and imaging features, individualized regimens can not only ensure safety, but also improve cognitive protection effects, such as virus‐mediated SIRT1 expression region‐specific regulation, precise administration of small molecule activators or inhibitors [115, 160, 173, 174, 175, 176, 177, 178] (Figure 6). The role of SIRT1 regulation in HD is significantly environment‐dependent and may vary depending on disease stage, cell type, and the balance between transcriptional stress and protein homeostasis stress.

## 5 Strategies to Modulate SIRT1

Given the multi‐target effects of SIRT1 in energy metabolism, inflammatory responses, and neurodegenerative diseases, precise modulation of its activity has become a critical research focus for the prevention and treatment of cognitive impairments. Therefore, various strategies aimed at modulating SIRT1 activity, including small molecule compounds, gene‐based approaches, and lifestyle interventions, can restore metabolic and protein homeostasis balance in cognitive impairment. Each of these strategies possesses distinct translational advantages and limitations (Table 4).

**TABLE 4:** Strategies for modulating SIRT1 activity.

Strategy	Representative approach	Primary mechanisms	Translational relevance	References
Small‐molecule activators	Resveratrol, SRT1720	Enhancement of deacetylase activity, anti‐inflammation, and autophagy	Animal and early clinical support	[10, 161, 179, 180, 181, 182, 183, 184, 185]
Small‐molecule inhibitors	EX527	Context‐dependent SIRT1 inhibition	Potential benefit in HD	[141, 186]
Lifestyle interventions	Caloric restriction, exercise	Increase NAD^+^ and activate AMPK–SIRT1 axis	Safe adjunctive strategy	[158, 162, 187, 188, 189, 190]
Gene‐based approaches	AAV‐mediated SIRT1 modulation; CRISPR/Cas‐based regulation	Region‐ or cell type‐specific regulation of SIRT1 expression/activity	High precision potential but limited by delivery, safety, and long‐term control	[173, 174, 176, 177, 178]

### 5.1 Small‐Molecule Agents: Activators and Inhibitors

#### 5.1.1 SIRT1 Activators

Resveratrol has frequently been described as a SIRT1‐activating compound, but its status as a direct enzymatic activator remains controversial. Biochemical studies using native substrates have reported that resveratrol does not directly increase SIRT1 catalytic activity and that some early activation signals depended on fluorophore‐containing assay substrates [179]. Its neuroprotective effects may instead involve indirect and dose‐dependent mechanisms, including AMPK activation, changes in cellular NAD^+^ availability and the NAD^+^/NADH ratio, and downstream engagement of SIRT1‐dependent pathways [180]. Within this framework, resveratrol has been reported to reduce Aβ‐related pathology and improve cognitive performance in experimental models, with limited but suggestive evidence from early clinical studies [181]. It may also influence the miR‐134/CREB/BDNF pathway, neuroinflammation, microglial states, and hippocampal network function [10, 182, 183, 184]. Therefore, resveratrol is better presented as a pleiotropic metabolic modulator that can engage SIRT1‐associated signaling rather than as an unequivocal direct SIRT1 agonist.

Building on this, synthetic small‐molecule SIRT1 activators such as SRT1720 have been developed with greater potency and stability than resveratrol [161]. SRT1720 can increase SIRT1 expression, activate AMPK and the Nrf2/heme oxygenase‐1 (HO‐1) antioxidant pathway, and suppress NF‐κB‐mediated neuroinflammation, thereby improving learning, memory, and cognitive performance. It can also reduce oxidative stress and lower expression of apoptotic proteins such as caspase‐3, providing multi‐layer neuroprotection [140, 161]. These findings suggest that SIRT1 activators act through network‐level, multi‐target regulation and remain an important direction for drug development in neurodegenerative diseases. However, early biochemical studies questioned whether SRT1720 directly activates SIRT1 with native substrates and reported activity at additional receptors, enzymes, transporters, and ion channels [185]. Thus, its neuroprotective effects should be interpreted with attention to dose, assay design, pharmacokinetics, and possible SIRT1‐independent actions.

#### 5.1.2 SIRT1 Inhibitors

SIRT1 inhibitors may exert different, and sometimes opposite, effects across neurodegenerative diseases, which makes their therapeutic value highly context dependent. In AD and PD models, suppression of SIRT1 generally aggravates core stress‐related phenotypes: mitochondrial biogenesis becomes less stable, p53/FOXO‐associated pro‐apoptotic signaling is enhanced, and inflammatory as well as oxidative stress pathways become stronger, together accelerating pathological progression [140, 186, 191]. Loss of SIRT1 activity tends to eliminate an important residual protective mechanism when metabolic reserves and redox balance are impaired. But HD may not be in this mode. Several HD model studies have shown that the selective SIRT1 inhibitor EX527 can reduce striatum atrophy, alleviate mHTT‐related transcription disorders and protein aggregation, and improve behavioral and cognitive outcomes [141]. This suggests that the role of SIRT1 depends on disease stage and cellular microenvironment, so the same intervention may have opposite effects in different situations.

Another critical issue is interference among anti‐aging enzymes. Available evidence indicates that simultaneous inhibition of SIRT1 and SIRT2 can synergistically suppress NF‐κB signaling and reduce inflammation induced by microglia, suggesting that multi‐target strategies may be more effective than single‐target approaches [140]. From a translational perspective, SIRT1 inhibition alone is unlikely to achieve the desired therapeutic effect. Selective and carefully tailored inhibition should be implemented based on disease type, disease stage, and the broader sirtuin network. Ideally, this should be guided by biomarkers to demonstrate whether the inflammation‐metabolic balance is shifting in the desired direction. Importantly, the reported synergistic suppression of NF‐κB by combined SIRT1/SIRT2 inhibition is supported mainly by cell‐based and in vitro evidence summarized in the cited literature; robust confirmation of the same synergy in neurodegenerative in vivo models remains insufficient. This limitation should be addressed before proposing combined inhibition as a translational strategy.

### 5.2 Gene Editing and Gene Therapy Strategies

Although current gene‐therapy efforts in the central nervous system often focus on disease‐causing mutations, the same delivery platforms may also support spatiotemporally controlled modulation of SIRT1. Rapid advances in genome engineering now allow increasingly precise manipulation of SIRT1. Clustered regularly interspaced short palindromic repeats/CRISPR‐associated protein 9 (CRISPR/Cas9) has been used to generate SIRT1 knockout models, providing a clear way to examine how SIRT1 influences metabolism, stress resistance, and neurodegeneration in vivo [174, 175]. Beyond gene knockout, newer base‐editing and epigenome‐editing methods can regulate SIRT1 transcription without changing its coding sequence, offering a more graded form of control [176, 177].

In recent years, CRISPR/Cas9 technology and adeno‐associated virus (AAV) vector systems have received broad attention in the treatment of neurodegenerative diseases, especially in gene correction strategies for disorders such as HD and AD. Through the gene‐editing capacity of CRISPR/Cas9, pathogenic mutations may be directly repaired, including errors in the mHTT gene, which may help delay disease progression [178]. At the same time, the AAV system has become an important tool for gene delivery, especially for the delivery of CRISPR/Cas9 components to specific brain regions, which is mainly due to its low immunogenicity and close carrier characteristics [173].

In general, gene editing and gene therapy techniques have shown significant potential to achieve SIRT1 regulation by targeting specific neural circuits at appropriate times and with appropriate intervention intensity. However, there are still many issues that require rigorous and systematic evaluation before widespread clinical application, such as long‐term safety, off‐target effects, immune responses, and regional differences in the brain. In addition to direct gene editing or viral‐vector approaches, non‐coding RNAs provide another potential level of SIRT1 regulation. In particular, microRNAs that target SIRT1 expression may offer a means to fine‐tune pathway activity, although tissue specificity, delivery efficiency, and off‐target regulation will need to be considered [156, 192].

### 5.3 Lifestyle Interventions: Diet and Exercise

CR and IF improve systemic metabolic flexibility and can engage AMPK–NAD^+^–sirtuin signaling in peripheral tissues. Evidence that these interventions directly and consistently increase SIRT1 abundance or activity in human neural tissue is less definitive because brain measurements are difficult, intervention protocols differ, and many observations derive from animal models or indirect biomarkers. It is therefore more appropriate to state that CR and IF may influence cerebral SIRT1‐related pathways through changes in energy status, ketone metabolism, insulin sensitivity, NAD^+^ availability, and peripheral‐to‐central signaling, rather than assuming a uniform direct increase in neural SIRT1. In experimental models, these adaptations have been associated with reduced oxidative stress and improved cognitive outcomes [187].

Exercise shows a similar regulatory effect. Evidence from both animal and human studies indicates that physical activity can increase SIRT1 levels and/or activity in blood and in cognition‐related brain regions such as the hippocampus. Once activated, SIRT1 can promote hippocampal BDNF expression, suppress neuroinflammation, and reduce Aβ‐related burden, thereby supporting synaptic plasticity and learning–memory performance [188]. In AD mouse models, regular exercise has been associated with higher brain SIRT1 levels and less cognitive impairment [158], and population studies report that higher physical activity correlates with increased peripheral SIRT1 alongside improved cognitive scores [162].

Taken together, lifestyle strategies based on dietary regulation and regular exercise provide a low‐cost and low‐risk way to increase SIRT1 activity over the long term. Rather than replacing pharmacological or gene‐based interventions, they may serve as a stable baseline that improves the metabolic environment, which often influences whether SIRT1‐targeted therapies are beneficial, ineffective, or potentially detrimental. Exercise may additionally influence SIRT1‐related brain responses through myokine‐mediated inter‐organ signaling [189]. CR and IF can engage energy‐sensing pathways while reducing inflammatory and oxidative stress relevant to neurodegeneration [190]. These adaptations may act as a form of metabolic priming that complements pharmacological SIRT1 modulation rather than functioning as an isolated treatment.

## 6 Future Directions

Current research is shifting from single‐pathway analyses toward system‐level integration. Multi‐omics studies have positioned SIRT1 at the core of protein–metabolite interaction networks, with its activity closely correlated to cognitive phenotypes. These findings reinforce the concept that SIRT1 represents a crucial target for maintaining cerebral metabolic homeostasis and delaying cognitive decline. Concurrently, other members of the deacetylase family, including SIRT3 and SIRT6, are gaining increased attention for their roles in energy metabolism and neuroinflammation. For example, studies have reported that SIRT6 modulates mitochondrial dynamics through the STAT5–PGAM5–Drp1 signaling axis, ameliorating cognitive impairment—a finding that suggests functional synergy within the deacetylase family.

From a translational medicine perspective, interventions such as caloric restriction, exercise training, and small‐molecule activators have demonstrated the potential to enhance deacetylase activity and improve cognitive scores in patients with mild cognitive impairment in clinical settings. However, establishing optimal interventional strategies remains challenging, and the correlation between peripheral biomarkers and cerebral pathological changes warrants further elucidation. A major translational barrier is the gap between robust effects in experimental models and the more variable responses observed in humans [193]. Limited brain bioavailability remains an important obstacle for pharmacological SIRT1 strategies [194]. Difficulty achieving cell‐type‐specific modulation, disease heterogeneity, insufficient biomarkers for patient stratification, and possible systemic effects further complicate long‐term SIRT1‐targeted therapy. The context‐dependent effects observed in HD further support biomarker‐guided strategies and more selective delivery platforms, including cell‐type‐ or brain‐region‐targeted vectors and nanoparticles [195].

Future research is expected to utilize single‐cell sequencing, spatial omics, and related technologies to evaluate the safety and efficacy of deacetylase modulation with high resolution across different cell types and brain regions. Furthermore, exploring combinatory strategies involving Aβ‐targeted and Tau‐targeted therapies may pave the way for developing individualized and more precise therapeutic approaches. Combination approaches may also be useful, particularly when the AMPK–SIRT1–PGC‐1α axis is targeted together with parallel inflammatory and proteostatic pathways [54]. Context‐dependent mediators such as PGE2 illustrate why the timing, dose, and disease stage of multi‐target interventions remain important considerations [196].

## 7 Conclusions

As an NAD^+^‐dependent deacetylase, SIRT1 regulates the activity of transcription factors and the functions of proteins, making it a core regulatory factor for metabolic homeostasis, inflammation control, and neural plasticity. This provides a systematic framework for understanding the maintenance and decline of cognitive function.

Across neurodegenerative diseases, SIRT1 intersects with key pathological nodes: Aβ/tau pathology in AD, proteostasis imbalance and mitochondrial dysfunction in PD and HD, and broader stress–inflammation coupling. Accordingly, SIRT1‐targeted strategies—including activators, inhibitors, gene‐based approaches, and lifestyle interventions—have shown therapeutic promise. However, these benefits are unlikely to be uniform across diseases and may depend on disease stage, brain region, cell type, and intervention intensity. The central goal for future research is to achieve moderate, precise control of SIRT1 activity. With support from multi‐omics, improved delivery technologies, and rational multi‐target combinations, SIRT1‐centered strategies may be better positioned for clinical translation into individualized therapies. However, most of the existing evidence is still at the preclinical stage, and the direction and intensity of SIRT1 regulation may depend on the disease stage, brain region, cell type, and intervention intensity.

## Author Contributions

**Jiabin Duan:** investigation, data curation, writing – original draft. **Jiaqi Liu:** investigation, data curation, writing – original draft. **Qingsheng Meng:** investigation, data curation, formal analysis, writing – review and editing. **Jiahao Liu:** investigation, data curation, formal analysis, writing – review and editing. **Sitian Yang:** investigation, data curation, formal analysis, writing – review and editing. **Fang Zhou:** conceptualization, supervision, writing – review and editing. **Jing Cao:** conceptualization, supervision, writing – review and editing, funding acquisition.

## Funding

This work was supported by the National Natural Science Foundation of China, Grant/Award Number: 82371237; and Program for Innovative Research Team in Universities of Henan Province, Grant/Award Number: 22IRTSTHN028.

## Ethics Statement

The authors have nothing to report.

## Consent

The authors have nothing to report.

## Conflicts of Interest

The authors declare no conflicts of interest.

## Data Availability

Data sharing not applicable to this article as no datasets were generated or analyzed during the current study.
